# Rare bilateral corneoscleral perforation secondary to ocular tuberculosis: a case report and clinical insights

**DOI:** 10.1186/s12348-025-00472-y

**Published:** 2025-03-05

**Authors:** Justin Dreyer, Lauren Kiryakoza, Jonathan Tijerina, Thomas Albini, Guillermo Amescua

**Affiliations:** Bascom Palmer Eye Institute, 900 NW 17 St, FL Miami, 33136 USA

**Keywords:** Ocular tuberculosis, Necrotizing scleritis, Immune-mediated uveitis, Anterior segment ocular coherence tomography (OCT), Ocular ultrasound

## Abstract

**Background:**

This case represents a rare presentation of bilateral corneoscleral perforation secondary to presumed ocular mycobacterium tuberculosis infection with the goal of reporting a case of bilateral corneoscleral perforation in the setting of a positive interferon-γ release assay (IGRA) test.

**Findings:**

A 27-year-old patient presented with five months of redness, worsening eye pain, and five days of decreasing vision. Visual acuity (VA) was counting fingers bilaterally. Intraocular pressure (IOP) was 10 mmHg and 19 mmHg in the right and left eye, respectively. A slit lamp examination disclosed conjunctival injection, corneal haze, bilateral mutton-fat keratic precipitates, and a hypopyon in both eyes. The right eye had a 1.5 mm × 1.5 mm Seidel-positive corneal perforation with uveal prolapse. Similarly, the left eye had a 0.5 mm × 0.5 mm Seidel-negative inferior corneal perforation with uveal plugging. The chest X-ray showed a left-sided pleural effusion.

**Conclusion:**

Computed tomography (CT) of the face and sinuses showed bilateral circumferential globe thickening. IGRA was positive. All other rheumatologic and infectious workups were negative, including HIV, ACE, ANA, ANCA, CRP, anti-scleroderma antibody and HCV. The patient was treated with intravenous methylprednisolone and seven months of rifampin, isoniazid, pyrazinamide, and ethambutol. This workup shows the rare bilateral corneal involvement of ocular tuberculosis.

## Introduction

Mycobacterium tuberculosis (MTB) is an acid-fast gram-positive bacillus that infects 10 million people every year, with an estimated 33% of the world’s population infected [1]. The Centers for Disease Control and Prevention (CDC) estimates up to 13 million are infected in the United States [1]. Worldwide, the highest prevalence of latent tuberculosis is in Africa [2]. Ocular MTB has an unclear pathophysiology, and it can infect any ophthalmic tissue. Current diagnostic techniques for ocular MTB are limited due to the rarity of the disease and the difficulty culturing MTB due to its fastidious nature [3]. The combination of clinically recognized ocular MTB presentations and positive immunological findings through purified protein derivative skin tests (PPD) or (IGRA) blood tests generally support the diagnosis of ocular MTB [4]. We present a case of MTB-associated necrotizing keratosclerouveitis complicated by corneoscleral perforation and uveal prolapse.

## Case report

**Fig. 1:**
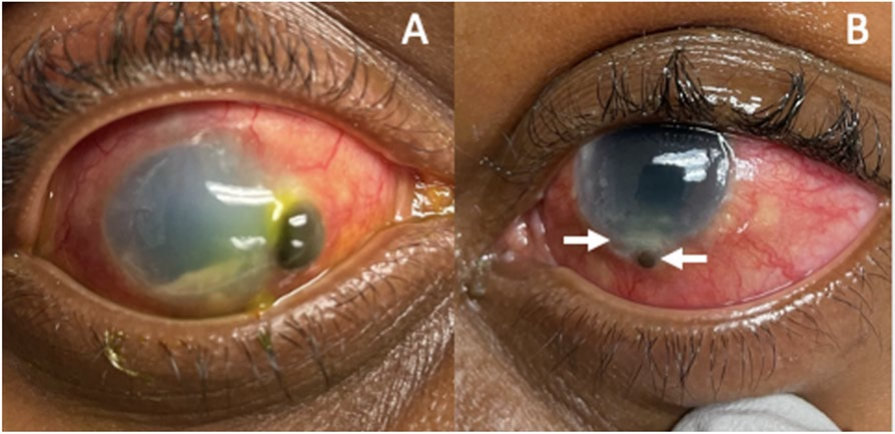
**A**: An external photo of the right eye shows pronounced conjunctival injection, diffuse corneal edema and haze, nasal corneal perforation with uveal plugging and a hypopyon. **B**: An external photo of the left eye shows diffuse conjunctival injection, inferior corneal edema with perforation and uveal plugging. A small hypopyon is present, though difficult to visualize with external photography

**Fig. 2:**
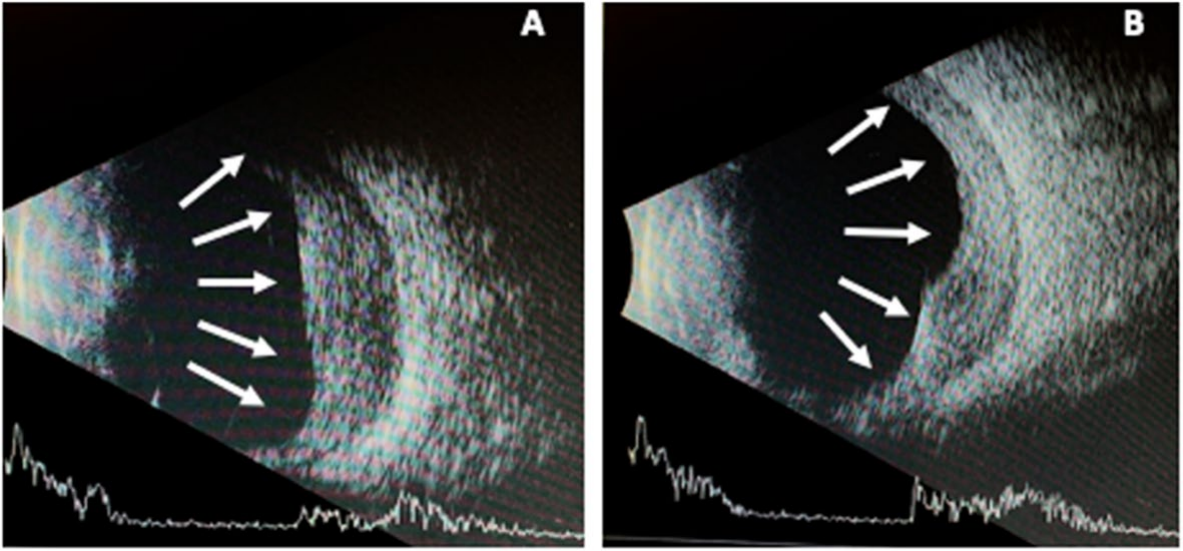
**A**: B-scan ultrasound of right eye showing diffuse choroidal thickening indicated by arrows. **B**: B-scan ultrasound of left eye showing diffuse choroidal thickening indicated by arrows

A 27-year-old female from the Caribbean presented with 5-months of persistent eye redness and pain and five days of decreasing vision in both eyes. On presentation, the right eye had diffused conjunctival injection, nasal corneal perforation with uveal prolapse that was Seidel positive, granulomatous keratic precipitates (KP), and a hypopyon. The left eye had an inferior corneal perforation with uveal plugging that was Seidel negative and a hypopyon (Fig. 1). The best corrected visual acuity (BCVA) was counting fingers in both eyes. Intraocular pressure was 10 mmHg and 19 mmHg in the right and left eye, respectively. There was no view to the posterior pole in either eye. B-scan ultrasound revealed severe diffuse choroidal thickening bilaterally (Fig. 2).

**Fig. 3:**
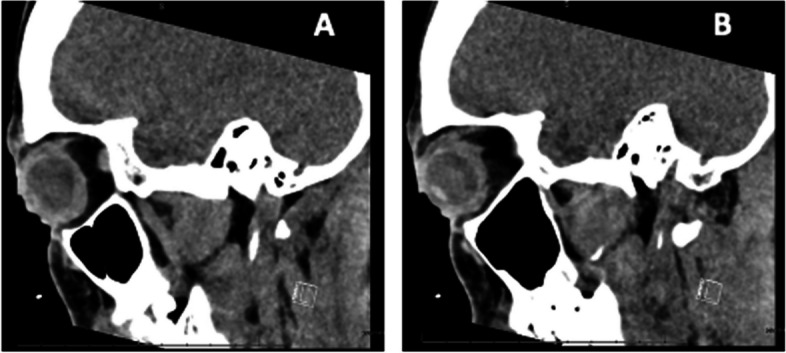
**A**: CT of the right eye shows circumferential thickening of the walls of the globe **B**: CT of the left eye also demonstrates circumferential thickening the walls of the globe

The patient was treated with corneal gluing and a bandage contact lens (BCL) in both eyes and admitted to the hospital for infectious and inflammatory diagnostic work-up. On day 1, the patient received broad-spectrum IV antibiotics (vancomycin, piperacillin, and tazobactam) and methylprednisolone. Chest X-ray showed a small left pleural effusion. CT chest found no pulmonary lesions. CT face and sinus showed non-specific circumferential globe thickening (Fig. 3). The patient underwent a comprehensive infectious and rheumatological workup. Blood cultures and P-ANCA, C-ANCA, HIV, and HSV-2 serologies were negative. The ESR was normal. The IGRA (TB1 5.77 and TB2 9.11 IU/mL) and HSV-1 IgG were positive. Treatment for presumed ocular MTB infection was initiated with rifampin (600 mg daily), isoniazid (300 mg daily), pyrazinamide (1000 mg daily), and ethambutol (800 mg daily) (RIPE). The methylprednisolone was started at 40 mg daily for 1 month then tapered to 30 mg daily then by increments of 5 mg every week.

**Fig. 4:**
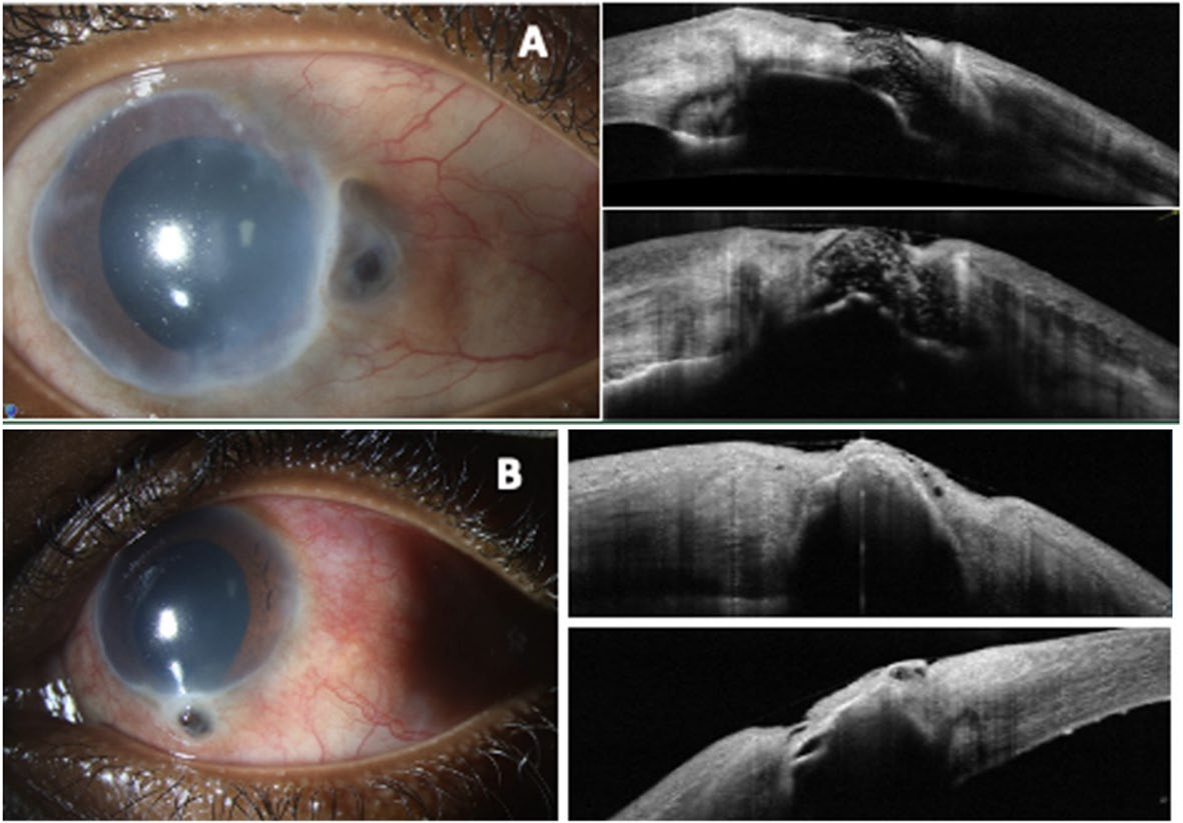
**A**: Right eye slit lamp photo demonstrates improved conjunctival injection and corneal edema, nasal corneal haze, stable uveal plugging of the prolapse site and a formed anterior chamber. The Anterior Segment OCT shows a hyporeflective lesion at the corneoscleral limbus likely reflecting uvea. **B**: Left eye slit lamp photos shows improved conjunctival injection, stable inferior uveal prolapse. The Anterior Segment OCT shows a hyporeflective lesion prolapsing into the corneoscleral limbus, likely reflecting uvea

After completing 7-months of RIPE treatment, the vision was hand motions OD and 20/40 OS. At the most recent follow-up, two years after the initial presentation, the visual acuity was hand motions OD and 20/20 OS. The anterior segment inflammation resolved. The fundus exam showed optic nerve pallor, attenuated vessels, and an attached retinal periphery with choriorteinal scarring. The slit lamp exam of the right eye showed diffuse corneal haze nasally and deep neovascularization (Fig. 4). The slit lamp exam of the left eye showed diffuse mild corneal haze (Fig. 4). Both Anterior Segment OCTs’ of both eyes revealed a hyporeflective lesion at the corneoscleral limbus, likely representing uveal tissue, with evidence of prolapse into the limbus (Fig. 4). There were no recurrent episodes of keratosclerouveitis (Fig. 4).

## Discussion

Ocular MTB can manifest in a variety of ways. The most common presentations are granulomatous anterior uveitis and posterior uveitis [5]. Choroidal granulomas, optic nerve granulomas, occlusive retinal vasculitis, and multifocal serpiginous-like choroiditis are frequently reported posterior manifestations [6, 7]. Generally, if the cornea is infected, it is considered a primary ocular site of infection with direct contact with the eye and will present phlyctenular keratoconjunctivitis, an inflammatory nodule at the limbus, or interstitial keratitis [5].

Bilateral presentation of ocular MTB is infrequent, ranging from just one out of 66 patients in one study to 30% in another [8, 9]. In a study of 66 patients with ocular MTB, only one patient had a bilateral presentation[8]. Among the rare bilateral presentations, the most common diagnosis was anterior uveitis [10, 11]. There have been other reported of similar corneal perforations but were rare occurrences [12, 13].

Corneal perforations often result from infectious keratitis, where the production of collagenolytic enzymes and inflammatory mediators leads to excessive degradation of corneal collagen [14, 15]. Elevated matrix metalloproteinases (MMPs), particularly MMP-2 and MMP-9, play a central role, with their overexpression driven by both microbial and nonmicrobial inflammatory mechanisms [15]. This sustained MMP activity contributes to significant corneal destruction, thinning, and perforation, as seen in bacterial, herpetic, fungal, and acanthamoeba infections [14, 15].

Ocular MTB is typically diagnosed clinically in conjunction with other supporting evidence such as interferon-gamma release assays and chest imaging [5, 12]. Direct sampling of ocular tissue is most feasible when infection is suspected in easily accessible tissue, such as the conjunctiva [16]. Diagnosis via direct sampling of MTB from uveal tissues or retinal tissues is more invasive and not commonly performed [17].

The Collaborative Ocular TB Score (COTS) score is a low-cost diagnostic tool that takes into account certain factors to diagnose MTB, including positive MTB skin tests or IGRA, positive chest imaging, origin from a MTB-endemic country [4]. It also considers if the clinical presentation is a generally recognized presentation of ocular MTB, such as multifocal vasculitis, tuberculoma, or serpiginous-like choroiditis [4]. Our patient’s COTS score was 4 [18]. A score of 4 or higher means 61–80% of experts would consider initiating anti-tubercular therapy [19]. Our patient was from an MTB-endemic country. The diagnosis of ocular MTB is typically only rendered when other pulmonary findings and evidence of MTB exposure is present [4]. There is a paucity of reports of ocular MTB presenting as keratitis. In one case, a patient with a unilateral MTB corneal ulcer with a positive IGRA test and granulomatous sequelae on chest CT was given antitubercular treatment and had resolution of the corneal ulcer in one month [20]. The authors postulated that the cornea, conjunctiva, and sclera involvement was a hypersensitivity reaction to tubercular protein rather than an active infection of MTB [20]. The COTS calculator, alongside established diagnostic protocols, is a useful adjunct tool for ocular MTB diagnosis. This approach not only reinforces the necessity of considering ocular tuberculosis, particularly in endemic regions, but also highlights the significance of recognizing atypical corneal manifestations as potential indicators of the disease. Such comprehensive assessment strategies are essential for the timely initiation of appropriate therapeutic interventions and improved patient outcomes.

## Conclusion

In summary, bilateral corneoscleral involvement of MTB is likely a rare presentation. The most common presentations of ocular MTB are granulomatous anterior uveitis and posterior uveitis [5]. Diagnosis of ocular MTB relies on supportive clinical findings and adjunct diagnostic testing with chest imaging and MTB skin test or IGRA blood testing. Recognition of bilateral corneal perforation secondary to infectious sources such as tuberculosis is important when rheumatologic entities are ruled out. Treatment with anti-tubercular therapy resulted in stabilization of the corneal pathology.

## Data Availability

No datasets were generated or analysed during the current study.
